# Now is not the time for isolationism: integrating global citizenship into higher education for the good of global health

**DOI:** 10.7189/jogh.08.020301

**Published:** 2018-12

**Authors:** Lee Stoner, Zachary Y Kerr, Dianne S Ward

**Affiliations:** 1 Department of Exercise and Sport Science, University of North Carolina, Chapel Hill, North Carolina, USA; 2 Department of Nutrition, Gillings School of Global Public Health, University of North Carolina, Chapel Hill, North Carolina, USA

The future of health care in northern hemisphere nations looks bleak. Within the United States (US), much of the recent political rhetoric has centred around “America First”, and health care reform to limit social medicine. Similarly, across Europe there is increased interest in far-right nationalism and a move away from inclusion. These movements encourage a culture of individualism and isolationism. Confounding the issue is the rise of “alternative facts”, diminishing interest in scientific inquiry, and the lack of meaningful and logical debate. With specific reference to health care, isolationism, coupled with diminishing interest in scientific rigour, will have long-term detrimental implications to the health of a nation. Arguably, higher education is well poised to play a role in encouraging meaningful debate. To achieve this end, it is important that higher education institutions incorporate opportunities for fostering Global Citizenship. The goal of this commentary is not to provide a comprehensive overview regarding these topics, but rather advocate for the continued discussion.

## Why is isolationism bad for the health of a nation?

Public health is a global issue; worldwide there were 57 million deaths in 2008, 63% of which were attributable to non-communicable diseases (NCDs) [1]. Despite the growing public awareness about NCDs and the consequences of related lifestyle choices, the incidence of NCDs continues to rise which creates a burden on global health care systems. An argument can be made that perhaps “personal responsibility” is not the answer, and that the focus should be on “global responsibility” [2]. However, “global responsibility” requires increased awareness from the individual. For example, an individual would need to be aware that his/her NCD not only affects his/her own quality of life, but also affects his/her family, community, and nation. For example, in the US, it has been estimated that the total cost of obesity, when limited to lost productivity, could be as high as US$ 66 billion annually [3]. Moreover, lifestyle choices associated with NCDs, including obesity, have been linked to climate change and subsequent biodiversity loss [4]. If individuals simply focus on themselves, the consideration of such community, national, and global costs are severely limited. Individual actions can and do have global consequences.

With regards to health care reform and the debate around minimizing social medicine, it is important to emphasize that the people being targeted by - and most likely to benefit from -social medicine initiatives (eg, low socioeconomic status, the elderly) also experience the greatest health disparities [5]. These disparities place disproportionate economic burden on societies, stall national productivity, and stress under-resourced health care services [6,7]. While limiting social medicine may result in short-term gains such as decreased health insurance premiums and tax dollar allotment, there is great potential for a long-term consequence of decreased community- and national-level overall health.

## What is global citizenship and why is it important?

There have been increasing calls, from both the political and academic arenas, to ensure the capacity of higher education students to think and act globally in order to effectively address political, social, economic, and environmental problems on a global scale.[8] This call should be extended to include global health, to encourage the perspective of health to be shifted from “personal responsibility” to “global responsibility”.

Although global citizenship is a highly contested and multifaceted term, three key dimensions are commonly accepted [9]: (1) global awareness (understanding and appreciation of one’s self in the world and of world issues), (2) social responsibility (concern for others, for society at large, and for the environment), and (3) civic engagement (active engagement with local, regional, national and global community issues). While this definition may be imperfect, in order for an institution of higher education to identify an appropriate pedagogical model, there must first be a philosophical platform on which to place the building blocks.

## How do we foster global citizenship (and global health)?

Given the complexity of the latent construct, global citizenship, there is unlikely to be a single pedagogical approach. Nonetheless, there is mounting evidence suggesting that international experiences provide powerful *dis*-orientating experiences, leading to deep reflection, critical analysis, and synthesis [10-13]. Support should be given to such international experiences, specifically those focused on global health issues. For example, students could use an international experience to investigate social medicine practices in other regions and how societal philosophy affects medical practice.

While international exposures can provide particularly powerful *dis*-orientating experiences, the ability to experience and critically reflect upon global issues can also occur within national and local contexts via engaging in dialogues within the diverse settings that universities, symposiums/conferences, and service learning opportunities provide. Further, the advent of social media and technology allows for such dialogues to occur despite physical distance. Because of the diversity in backgrounds, nationalities, and ideas, it is important for all active participants to engage in and promote cordial discourse that encourages independent thinking and discourages emotionally-driven and acerbic reactions.

Irrespective of whether the experimental opportunity is local, national or international, these opportunities must be married with appropriate pedagogical practice. One such model is Thornton’s [14] three Ds: Directing (first telling the students what to do, how to do it, and when it needs to be done), Discussing (challenging students with questions to guide discussion and illuminate the students’ biases, worldview, perspective, and attempts to challenge preconceived notions), and Delegating (encouraging the development of questioning from students themselves). Thus, integrating global issues is simply not accomplished by providing examples of research done in international settings, but rather, by using these settings to drive a dialogue focused on the barriers and facilitators that may parallel those and differ from research performed in local settings. This can also integrate discussion of such commonalities and differences that may exist at the individual, regional, and national levels. Last, discussions should culminate with opportunities to brainstorm solutions to minimize barriers and inequalities.

Finally, any discussion of higher education promoting global citizenship should also inherently encompass equity. Providing opportunities for only “privileged” segments of a population may further exacerbate inequities [15]. Opportunities that target groups lacking “privilege” are warranted in order to help ensure their presence in dialogue related to global citizenship and global health. For example, scholarships may assist in funding travel for first-generation students or students from low-income families.

**Figure:**
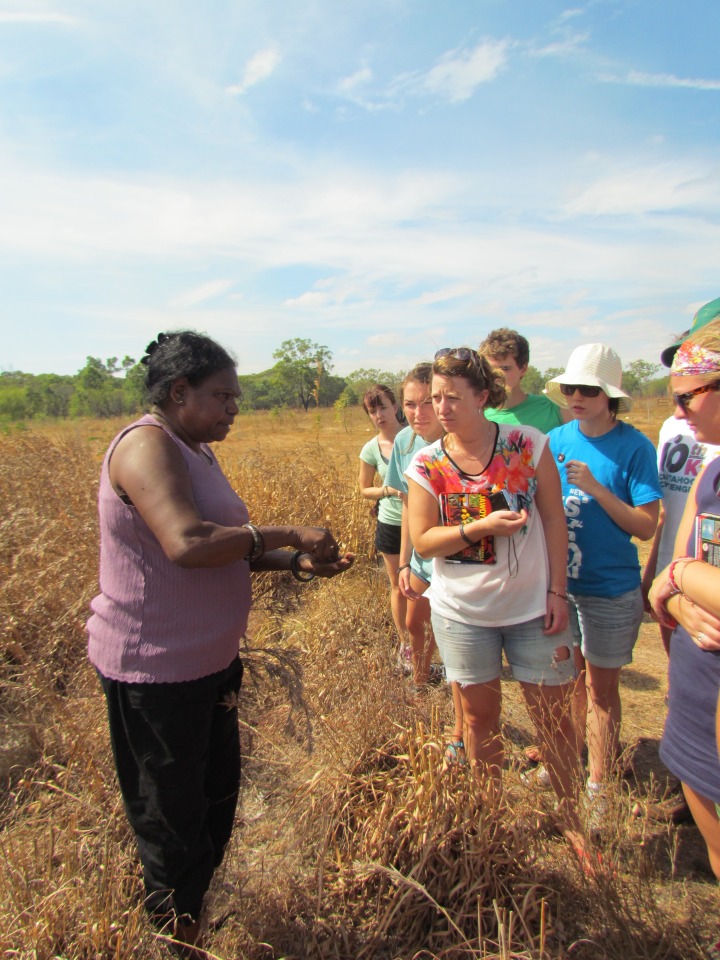
Photo: from the collection of Lee Stoner (used with permission)

## CONCLUSIONS

The growing trend in promoting isolationism in the northern hemisphere is compromising the health of nations. Focusing on the individual prevents a person from being cognizant of the local, national, and global implications of his/her own personal health. This ideological shift towards isolationism is also encouraging support of health care policies that emphasizes short-term gain yet results in long-term loss. These are not simple, black and white issues, and debate on such topics is needed. However, debate is being stymied by a lack of global awareness and the advent of “alternative facts”. Arguably, it is now more important than ever that institutes of higher education play a role by fostering global citizenship. A global citizenship competency, within the context of global health, should become an integral component of a university’s core curriculum.
